# Slow-Binding and Covalent HDAC Inhibition: A New Paradigm?

**DOI:** 10.1021/jacsau.4c00828

**Published:** 2024-10-31

**Authors:** Yasir S. Raouf, Carlos Moreno-Yruela

**Affiliations:** † Department of Chemistry, United Arab Emirates University, P.O. Box No. 15551 Al Ain, UAE; ‡ Laboratory of Chemistry and Biophysics of Macromolecules (LCBM), Institute of Chemical Sciences and Engineering (ISIC), School of Basic Sciences, École Polytechnique Fédérale de Lausanne (EPFL), CH-1015 Lausanne, Switzerland

**Keywords:** histone deacetylase, covalent inhibitor, slow-binding, inhibitor kinetics, cancer chemotherapy

## Abstract

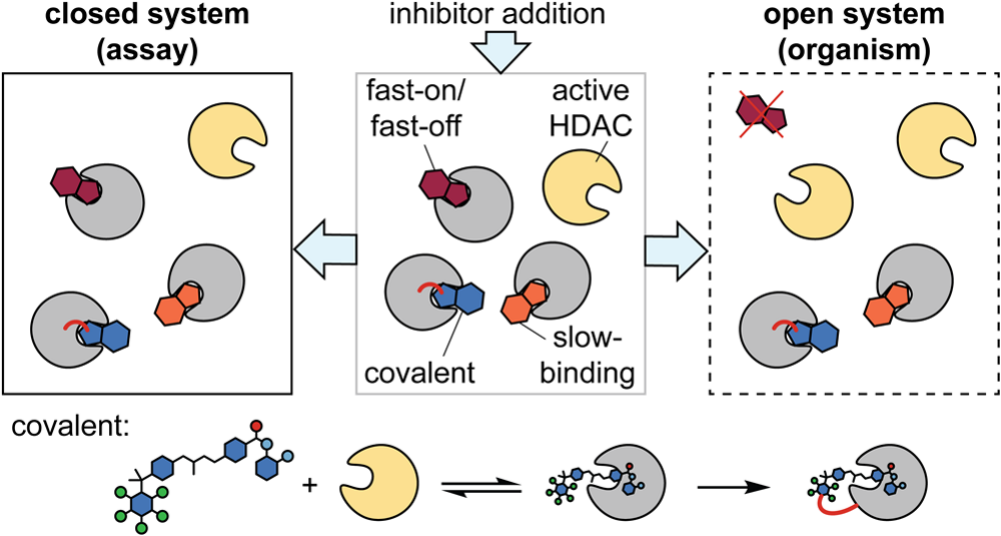

The dysregulated post-translational modification of proteins is an established hallmark of human disease. Through Zn^2+^-dependent hydrolysis of acyl-lysine modifications, histone deacetylases (HDACs) are key regulators of disease-implicated signaling pathways and tractable drug targets in the clinic. Early targeting of this family of 11 enzymes (HDAC1–11) afforded a first generation of broadly acting inhibitors with medicinal applications in oncology, specifically in cutaneous and peripheral T-cell lymphomas and in multiple myeloma. However, first-generation HDAC inhibitors are often associated with weak-to-modest patient benefits, dose-limited efficacies, pharmacokinetic liabilities, and recurring clinical toxicities. Alternative inhibitor design to target single enzymes and avoid toxic Zn^2+^-binding moieties have not overcome these limitations. Instead, recent literature has seen a shift toward noncanonical mechanistic approaches focused on slow-binding and covalent inhibition. Such compounds hold the potential of improving the pharmacokinetic and pharmacodynamic profiles of HDAC inhibitors through the extension of the drug–target residence time. This perspective aims to capture this emerging paradigm and discuss its potential to improve the preclinical/clinical outlook of HDAC inhibitors in the coming years.

## 1 Introduction – HDAC Structure and Function

Histone deacetylases (HDACs) are Zn^2+^-dependent hydrolases of protein ε-*N*-acetyllysine (Kac) modifications and of other acyl-amine groups. HDACs were first discovered to erase Kac post-translational modifications (PTMs) from histones,^1,2^ which determined their name and fundamental role in regulating DNA packing and gene expression. However, research over the past 30 years has uncovered a much wider network of modifications and biological processes regulated by HDACs, as well as deacylation-independent roles in gene regulation and cell signaling.^3−5^

**Figure 1:**
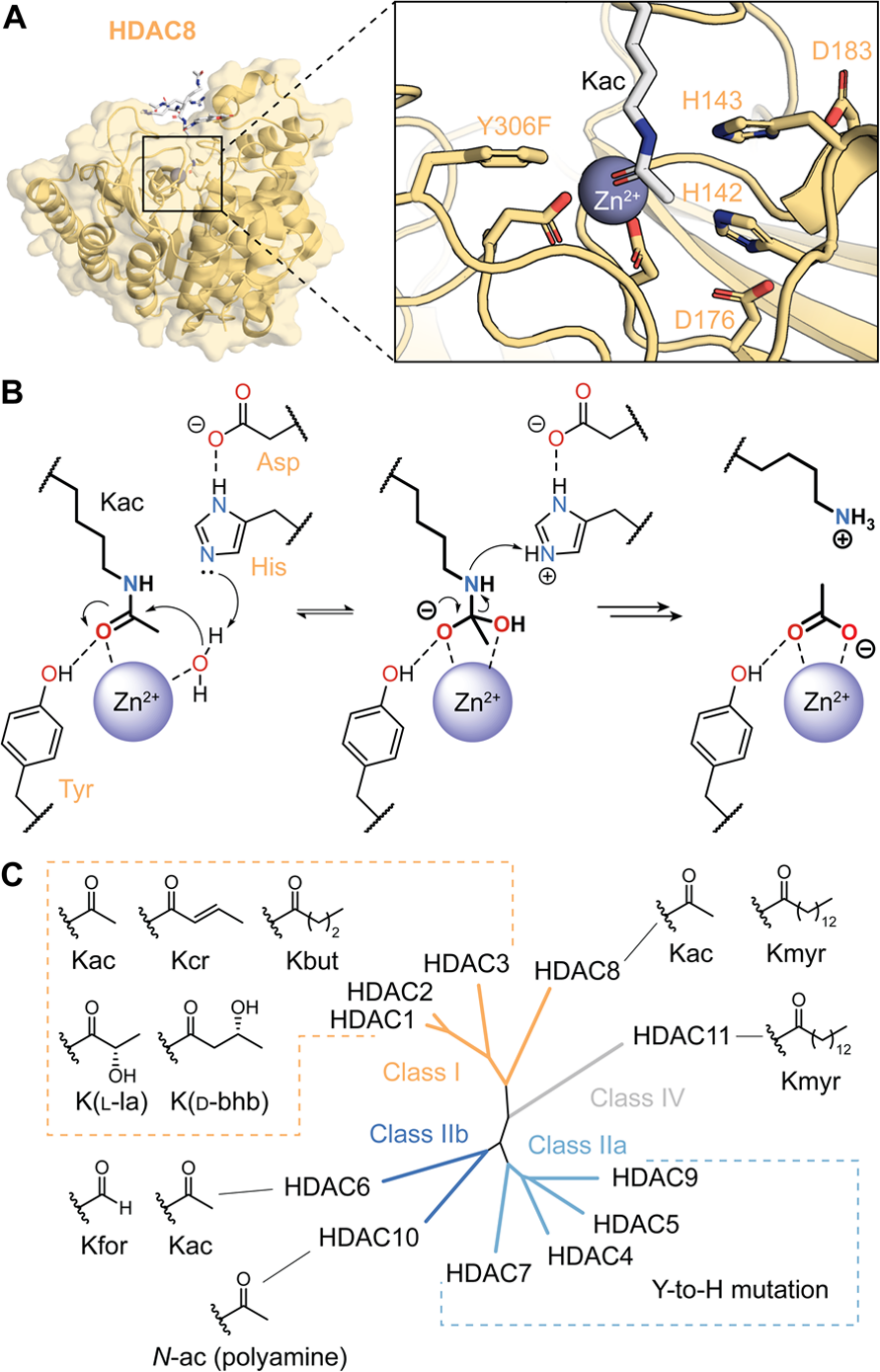
(a) Crystal structure of HDAC8 bound to a Kac substrate, with highlighted residues involved in catalysis (PDB 2V5W, HDAC8 has an inactivating Y306F mutation). (b) Summary of the mechanism of deacetylation by HDACs. (c) Phylogenetic relationship of the 11 human HDACs, and representative acyl substrates.

The structure of the HDAC catalytic domain belongs to the arginase-deacetylase superfamily, which spans multiple enzyme families across the tree of life.^4^ The α/β arginase-deacetylase fold in HDACs accommodates a Zn^2+^ ion within a hydrophobic tunnel, which coordinates to the acyl-amine substrate and catalyzes its hydrolysis (Figure 1A).^4,6^ After initial nucleophilic attack by a water molecule, the resulting tetrahedral intermediate is further stabilized by a Tyr residue within the catalytic tunnel, and two Asp-His pairs help catalysis through proton exchange (Figure 1B).^6^ The cavity where the Zn^2+^ ion sits varies in size and structure, as highlighted by the variety of acyl-lysine PTMs that different HDAC isozymes can accommodate (Figure 1C).^4^ In addition, certain HDACs feature a second cavity or “foot pocket” beyond the Zn^2+^ ion that has been employed for inhibitor development.^7,8^ The rim of the hydrophobic tunnel also displays isozyme-specific residues that are critical for their substrate preferences.^9^ Beyond this rim, HDACs present a variety of topologies and functionalities, which are often linked to their substrate recognition mechanisms and regulatory multiprotein complexes.^10,11^

There are 11 mammalian HDACs divided into 4 classes: class I, class IIa, class IIb, and class IV (Figure 1C). HDACs 1, 2, 3, and 8 (class I) are nuclear enzymes that fulfill the canonical role of histone deacetylation.^3^ In particular, HDACs 1–3 are the most active deacetylases in the nucleus and regulate chromatin structure and gene expression through modification of histones, chromatin remodelers and transcription factors.^12^ These three enzymes serve as the deacetylase module of a variety of multiprotein complexes with additional chromatin readers and modifiers.^11,13,14^ Thus, they are deeply involved in the epigenetic network of gene regulation. HDACs 1–3 also recognize less abundant histone PTMs such as crotonyl-lysine (Kcr)^15,16^ β-hydroxybutyryl-lysine (Kbhb)^17^ and lactyl-lysine (Kla),^18^ which have arisen as key modifications in disease due to their connection to metabolic imbalance (Figure 1C).^19,20^ In contrast with the other class I HDACs, HDAC8 is a less active histone deacetylase (*k*_cat_/*K*_M_ ∼ 50 M^–1^ s^–1^ on H3K9ac peptides,^21^ compared to ∼500 for HDAC1 and ∼150,000 for HDAC3/NCoR2)^18^ and has not been found to interact with nuclear multiprotein complexes. Instead, HDAC8 is a more specific deacetylase of histone complexes, chromatin-associated structural proteins and transcription factors.^12,22,23^ HDAC8 can also hydrolyze longer aliphatic and fatty acid-derived lysine PTMs.^24^

The class IIa HDACs 4, 5, 7, and 9 are nuclear proteins with marginal catalytic activity, due to a shared mutation of the catalytic Tyr residue to a His (Figure 1B).^25^ As a result, these proteins are considered to be readers of Kac marks that recruit other factors to chromatin.^26^ Class IIa HDACs feature large N-terminal extensions that include transcription factor-interacting domains, and phosphorylation sites that lead to nuclear export.^27,28^ Thus, they serve as regulatory switches in gene activation.^29^

HDAC6 and HDAC10 belong to class IIb HDACs, and they are highly efficient deacetylases of cytosolic targets (Figure 1C). HDAC6 has two catalytic domains: CD1 that targets C-terminal Kac modifications,^30^ and CD2 that serves as the main regulator of α-tubulin acetylation.^31,32^ HDAC6 also targets microtubule-associated proteins^33^ and can bind ubiquitylated targets through a C-terminal zinc-finger domain.^34^ HDAC10, on the contrary, does not target acyl-lysine PTMs (*k*_cat_/*K*_M_ > 200-fold lower on Kac substrates compared to HDAC1–3 and HDAC6)^35^ but rather functions as polyamine deacetylase.^36^ The respective substrate selectivity of HDAC6_CD1 and HDAC10 are dictated by specific mutations within the active side rim.

A conserved Leu residue is replaced by a “gatekeeper” K353 in HDAC6_CD1, which confers selectivity toward the C-terminal carboxylate, and by E272 in HDAC10, which interacts with the positively charged polyamine scaffold.^30,36^

Finally, the smallest isozyme and sole member of class IV in mammals is HDAC11.^37^ Even though HDAC11 is present in the nucleus and involved in gene regulation, its histone deacetylase activity is elusive. Instead, HDAC11 was found to remove fatty acid-derived PTMs such as myristoyl-lysine (Kmyr, *k*_cat_/*K*_M_ ∼ 15,000 M^–1^ s^–1^)^38^ for protein trafficking and cell signaling.^38−41^

As primary regulators of the cellular acetylome,^42^ HDACs play important roles at every stage of cellular function, including division, movement, adhesion, metabolism, and signaling.^43,44^ The deacetylation of histones and other chromatin-associated nuclear targets (e.g., BRCA1, NF-κB, STAT3) renders HDACs crucial regulators of chromatin structure and gene regulation. Importantly, HDACs regulate pro-apoptotic signals (e.g., p21, BAX), tumor suppressor and angiogenesis factors (e.g., p53., HIF1α), as well as biomarkers of cellular adhesion (e.g., CDH1). Thus, HDACs control neoplastic processes^45^ and their dysregulated activity is strongly liked to tumorigenesis and a variety of human cancers. Despite their general role in oncology, these enzymes have been mostly studied within the context of blood cancers revealing important roles in hematopoiesis, hematopoietic stem cell fate determination, and early stage differentiation.^46^ This historical link to hematology likely emanated out of the initial studies on erythroid leukemia cell differentiation by Friend, Lapeyre, and Bekhor,^47,48^ which ultimately led to the regulatory approval of SAHA (vorinostat) in cutaneous T-cell lymphoma (CTCL). However, HDAC targeting keeps holding promise in other types of cancer.^49^

Outside of oncology, the last 10 years have seen extensive research linking HDAC activity to alternative indications including neurodegenerative disease, metabolic disorders, cardiovascular disease, and viral infections.^50^ Dysregulated acetylation is a hallmark of neurological disease and by affecting synaptic plasticity, tau phosphorylation, and the expression of cognition-related proteins, HDACs have been widely linked with Alzheimer’s disease (AD).^51^ In addition, action on neuronal demyelination and the induction of cytosolic protein aggregates has specifically linked class IIb isozyme HDAC6 in Charcot-Marie-Tooth disease (CMT)—a severe neuropathy of the peripheral nervous system.^52^ The roles of HDACs in the transcription of muscle-specific proteins, autophagy, and microtubule stability, provided a link to Duchenne muscular dystrophy (DMD).^53^ Moreover, dysregulated HDAC activity related to glucose homeostasis, hepatic fibrogenesis, and adipogenesis has also linked these enzymes to metabolic disorders. Here, HDACs are primarily linked to diabetes mellitus, since multiple HDAC isotypes (HDAC1–3, HDAC6, HDAC11) regulate insulin signaling, metabolic switching, hepatic gluconeogenesis, β-cell apoptosis, and renal inflammation.^54^ In cardiac disease, HDAC9 has emerged as an important regulator of vascular health and atherosclerosis. This class IIa isozyme was found to play key roles in cholesterol transport, and the development of atherosclerotic lesions.^55^

Demonstrating the complex nature of epigenetic signatures within these multifactor chronic conditions, in cardiac hypertrophy, while specific isozymes can cause disease (e.g., HDAC2), other HDACs promote protective mechanisms (e.g., HDAC4).^56^ Finally, viral infections have been linked to deacetylase function with roles across viral entry, fusion, replication, latency, and release.^57^ A notable example is within human immunodeficiency virus (HIV), which is in dire need of novel and improved therapeutic modalities. In this regard, HDAC6 controls HIV-1 transcription and virion entry and release by affecting microtubule dynamics and cytoskeletal structural integrity. Ultimately, through control of cell-wide acetylation (and downstream signaling cascades), these enzymes are involved in a myriad of human diseases, and targeted epigenetic discovery efforts can provide immense benefit to millions of patients around the world.

## 2 Non-selective and Selective HDAC Inhibition

**Figure 2:**
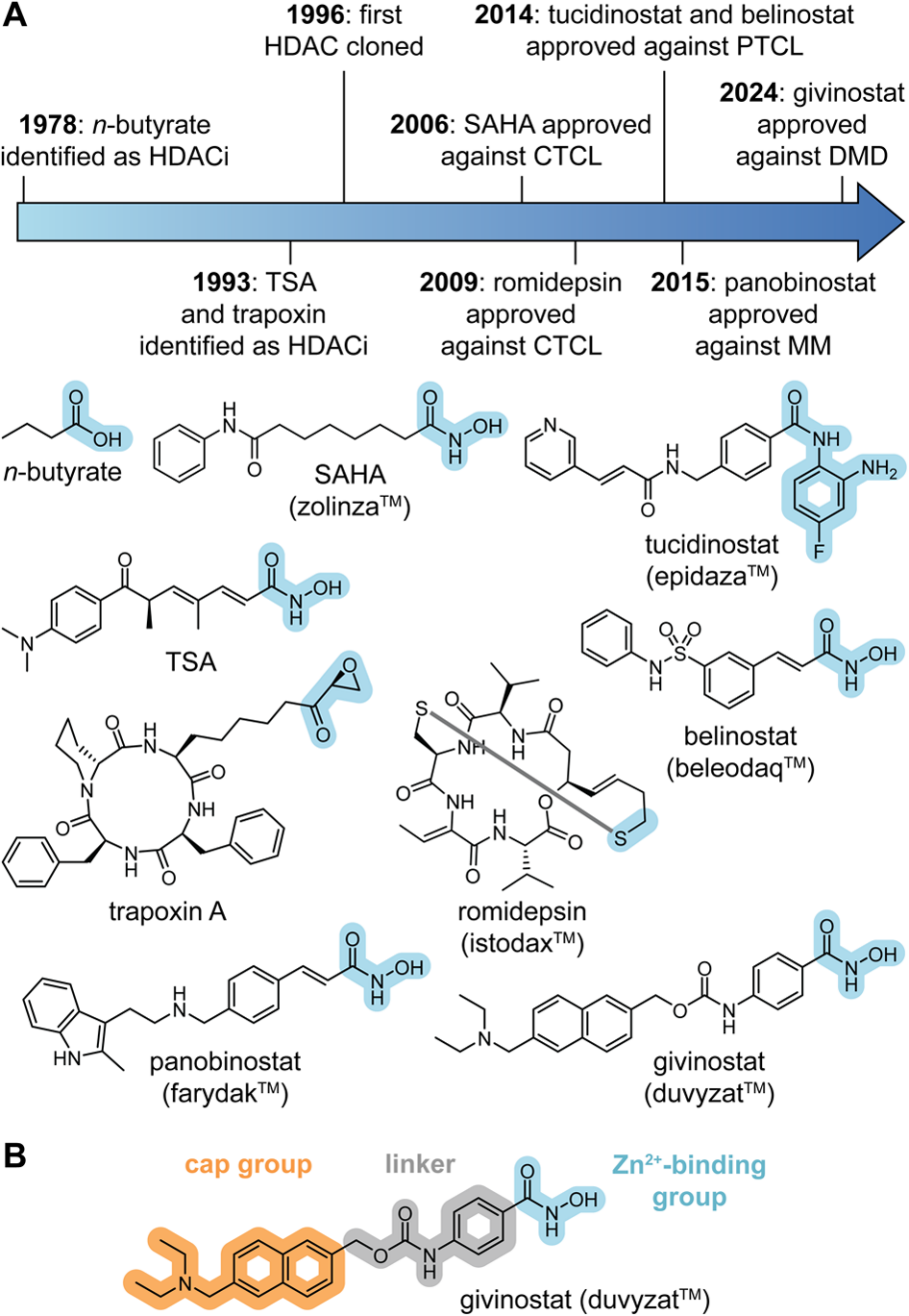
(a) Timeline and chemical structures of early HDACi identification and drug approvals. (b) Common pharmacophore of HDAC inhibitors.

As an approach to drug discovery, the targeted inhibition of HDACs to treat human disease is a relatively recent undertaking. While the initial link between histone PTMs and mRNA synthesis was reported in 1964 by Allfrey et al.,^58^ followed by studies using *n-*butyrate to affect histone acetylation and cell differentiation (Figure 2A), it is only in the last 30 years that pharmacological modulation of deacetylase enzymes has advanced into a viable clinical strategy.^59^ At the time of writing this, the field has reached over 1,250 articles per year on www.pubmed.gov (2023–2024), compared to 5 hits in 1990, with growing applications against various therapeutic indications (e.g., cancer, neurology, inflammation) and, more importantly, 6 drug approvals.

The discovery of early HDAC inhibitors (HDACi) was quite serendipitous, emerging from target-agnostic phenotypic screens of simple polar molecules and entirely unrelated research on antifungal antibiotics such as trichostatin and trapoxin (Figure 2A).^60^ While studying murine erythroleukemia cells, Friend et al.^47^ reported that the exposure to mM concentrations of dimethyl sulfoxide induced terminal differentiation and cell growth arrest. Marks et al.^61,62^ extended this observation to other carbonyl compounds including *N*-methylacetamide and N-dimethylformamide. Pursuing a predicted metal interaction, a series of bishydroxamic acids were found to be several times more potent than their amide counterparts. Subsequent structure–activity analyses exploring aromatic tail groups and varied methylene spacers ultimately led to the discovery of suberoylanilide hydroxamic acid (SAHA, Sloan-Kettering Institute for Cancer Research, Columbia University, Figure 2A).^63−65^ Due to its structural similarity to the bacterial metabolite trichostatin A (TSA)—a natural antibiotic and bona fide HDACi^1^—SAHA was confirmed as an inhibitor of HDACs. These findings, together with the isolation and cloning of the first HDAC in 1996^2^ and the first cocrystal structure of a bacterial deacetylase by Finnin et al.,^66^ spurred an outburst of interest in deacetylases as ligandable drug targets.

SAHA was found to cause up-regulation in acetylated substrates, influencing gene transcription and ultimately inducing cell cycle arrest and apoptotic cell death. This trend was observed across a wide panel of cancer cell lines.

Target engagement was also confirmed in follow-up crystallographic studies, clearly highlighting a bidentate coordination mode between the hydroxamic acid and the active site Zn^2+^ ion.^67−69^ In 2006, SAHA (zolinza, Aton Pharma/Merck & Co.) was approved for the treatment of CTCL. As the first targeted deacetylase inhibitor to enter the clinic, the success of SAHA spurred other clinical oncology studies, which led to several approvals primarily within the context of blood cancers. These included prodrug and natural product depsipeptide romidepsin (istodax, 2009) against CTCL, cinnamoyl hydroxamic acid belinostat (beleodaq, 2014) and 4-fluoro-2-aminobenzanilide tucidinostat (epidaza, China 2014), both against peripheral T-cell lymphoma (PTCL) and cinnamoyl hydroxamic acid panobinostat (farydak, 2015) in combination with bortezomib and dexamethasone against multiple myeloma (MM). Beyond cancer, the phenylhydroxamic acid givinostat (duvyzat, 2024) has recently received approval for the treatment of DMD as the first nononcology HDACi on the market (Figure 2A).^50^ If we consider the past decade, these breakthroughs have had a strong influence on all subsequent HDACi research - perhaps in a manner greater than other drug classes. Ultimately, these first generation molecules defined a lasting pharmacophore with repeated sets of privileged motifs and specific design strategies.

Standard HDACi are generally competitive acetyl lysine mimetics with 3 defining features: A Zn^2+^-binding group (ZBG), an elongated linker region, and a surface cap group (Figure 2B). The critical ZBG aims to coordinate the active site Zn^2+^ through mono- or bidentate modes. In most cases, this interaction represents the central driving force behind the observed potency. While ZBGs have advanced since the early days of sulfoxides, thiols, and simple carboxylates, the *N*-hydroxamic acid remains the most commonly used motif in HDAC literature. Despite known metabolic issues and a potential for idiosyncratic toxicities, this group persists for several reasons: a particularly superior affinity for Zn^2+^, simple synthetic routes, high polarity, improved aqueous solubility (as the rest of the molecule tends to be quite lipophilic), as well as precedence in literature (e.g., SAHA), and nature (e.g., TSA).^70^ Next in line is the *o-*aminoanilide group (often referred to as benzamide) seen in tucidinostat. While weaker as a Zn^2+^ chelator, this motif is uniquely selective for class I HDAC1–3 (owing to their larger active sites) and offers several additional benefits: improved systemic stability, slow-binding kinetics, and the capacity for synthetic derivatization. Outside of these two motifs, several novel ZBGs have also been recently reported. Notable examples include trifluoromethylketones, α-ketoamides, 2-(difluoromethyl)-1,3,4-oxadiazoles (DFMOs), and mercaptoacetamides.^71^

For the linker region, its extended structure spans the lysine tunnel (*d* = 6–10 Å) and has been largely explored using aliphatic hydrocarbons, vinyl extensions, and aromatic ring systems. Interestingly, recent studies have highlighted the value of linker choice, and heteroatom insertions to capture favorable intratunnel interactions.^72^ Lastly, the terminal surface cap sees the highest degree of structural variance and is generally the first design element optimized in structure–activity relationship (SAR) programs. This mainly involves the use of varied aromatics and heterocycles, or larger moieties inspired by trapoxin or apicidin. The surface cap is also commonly used to optimize physicochemical properties such as lipophilicity and solubility.

While this first wave of clinical HDACi set an important precedent, their widespread and long-term use was quickly restricted by recurring dose-limiting toxicities (e.g., thrombocytopenia, pyrexia, cardiac arrythmia, diarrhea, fatigue).^73^ These symptoms were mainly attributed to the indiscriminate inhibition of HDAC enzymes and cell-wide disruption of the acetylome. Without a selectivity scaffold, hydroxamic acids such as SAHA or panobinostat inhibit about half of the 11 HDACs. Moreover, with >300 Zn^2+^ metalloenzymes in the cell, hydroxamic acids often suffer from side effects due to off-targets. Strikingly, in a recent study, > 20 established hydroxamate-based HDACi were found to also inhibit metallo-β-lactamase domain-containing protein 2 (MBLAC2) - palmitoyl-CoA hydrolase with nM potencies.^74^ Furthermore, hydroxamic acids are also notoriously susceptible to phase I (reduction, hydrolysis) and phase II (glucuronidation) metabolic processes, with established links to mutagenicity and genotoxicity effects,^75^ and may also form reactive isocyanates through Lossen rearrangement in vivo.^75,76^ The acidity of the *N*-hydroxy group (p*K*_a_ 8.0–9.5, 25 °C) can also compromise oral bioavailability, intestinal permeability and effective distribution.^77,78^

Ultimately, poor safety profiles with unpredictable pharmacology and dose-limited clinical use sparked paradigm shifts into alternative design strategies to overcome these issues. These included the aim for more selective inhibitors, the use of alternative ZBGs, combination dosing and, later on, noncanonical inhibition mechanisms (discussed in the next section).^10,79^

**Figure 3:**
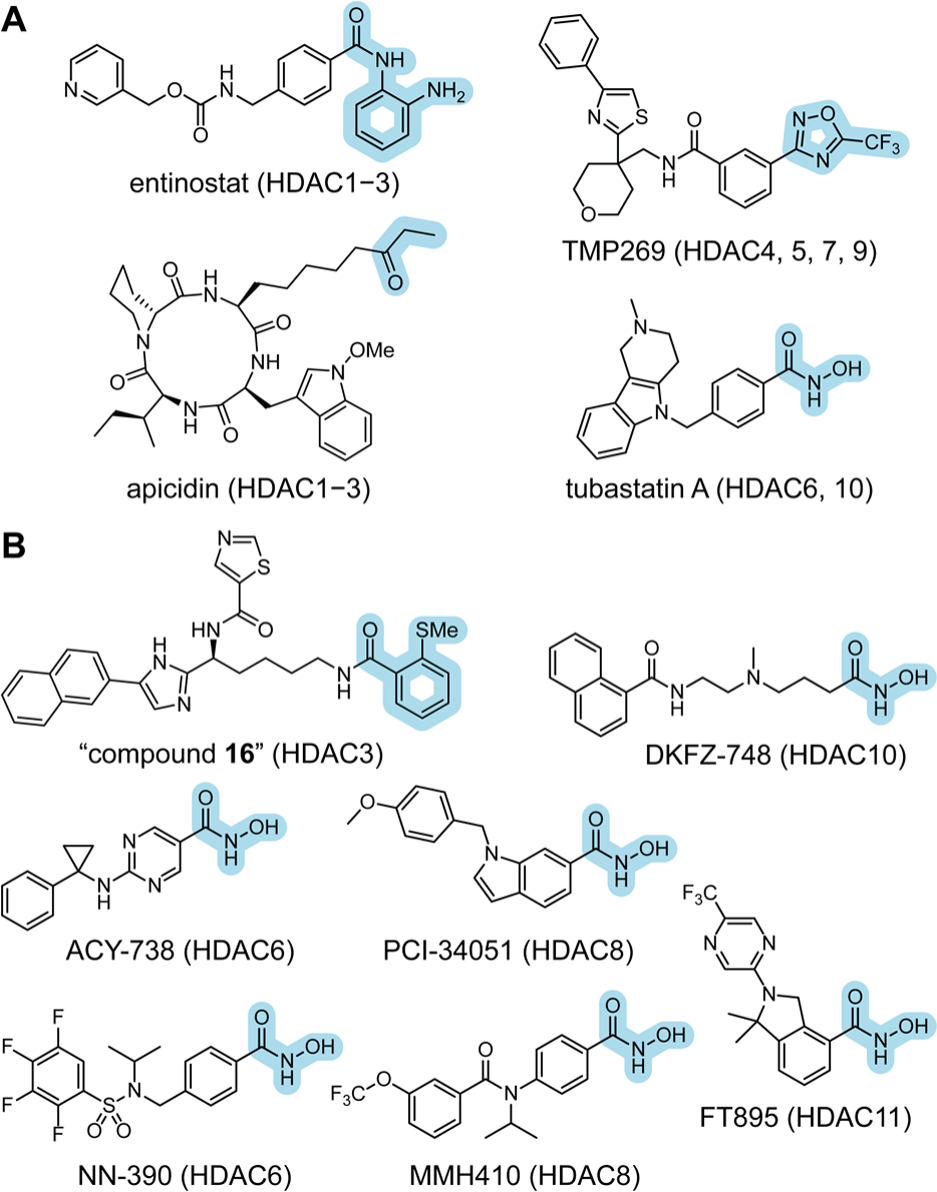
(a) Structure of selected class- and subclass-selective HDACi. (b) Structure of selected isozyme-selective HDACi.

Selective HDACi exploit subtle differences in protein structure to achieve discriminative target engagement. Strategies focus on either a single isozyme (e.g., HDAC6) or a subset of deacetylase enzymes (e.g., HDAC1–3). While HDACs are known to share high catalytic domain sequence identities (35–94%), the active site differences revealed by crystal structures have been targeted as selectivity filters via structure-based approaches.^80,81^ HDAC1–3 have a slightly larger active site that can accommodate *o*-aminoanilides, with HDACs 1 and 2 showing an additional L-shaped “foot-pocket” (*d* = 3–14 Å).^8^ Studies into HDAC6 revealed a rigid pair of Phe residues flanking the lysine tunnel,^80^ while a buried catalytic Tyr in HDAC8 forms a surface exposed L1-L6 cavity. The atypical “gatekeeper” residues of HDAC6 and HDAC10 (Lys in HDAC6_CD1 and Glu in HDAC10) have also been targeted for selective inhibition.^82^ In general, these strategies have been successful and provided class- and subclass-selective molecules against HDAC1–3 (entinostat), HDAC4, 5, 7, 9 (TMP269), or HDAC6, 10 (tubastatin A, Figure 3A) and compounds with more exquisite selectivity against HDAC3 (compound **16**),^83^ HDAC6 (ACY-738, NN-390, TO-317), HDAC8 (PCI-34051, MMH410), HDAC10 (DKFZ-748), and HDAC11 (FT895, Figure 3B).^84−92^ While useful as chemical probes within biological studies and preclinical animal models, these selective molecules have unfortunately not yet resulted in any significant clinical benefit. This highlights potential disconnects between in vitro selectivity profiles (derived from isolated inhibition activity assays) and subsequent in vivo pharmacology.

While targeting a single HDAC can be regarded as a safer strategy due to reduced toxicity, it can also lead to lower pharmacological potency. In fact, it has been posited that the clinical efficacy of the first generation of HDACi stems from the inhibition of multiple HDAC isozymes. Thus, it appears that a delicate balance between selectivity, toxicity, and potency must be struck to achieve full clinical potential, within a specific optimal target profile for each indication.

A second strategy to improve upon the first generation of HDACi was the exploration of novel ZBGs with improved pharmacokinetics and acceptable affinities for Zn^2+^ (Figure 3). While approved HDACi still rely on the hydroxamic acid, it is worth noting that these molecules have been long in development and reflect a time where this motif was in fact the best option. In hindsight, it appears that for the longest time, the field operated with a sense of complacency with respect to the Zn^2+^ ligand. It was common to install a hydroxamic acid at the start and focus on the optimization of the other structural motifs, without probing alternative ZBGs. This matter has been actively reconsidered in the past decade, with several useful strides to begin departing from the hydroxamic acid. *o*-Aminoanilides have been investigated extensively due to their added selectivity toward HDAC1–3 and potential for brain permeability,^93^ with the goal of addressing neurological diseases.

Similarly, trifluoromethyl heterocycles drew attention for targeting class IIa HDACs, which are often more difficult to target with hydroxamic acids (Figure 3A). A more recent example is the emergence of fluorinated oxadiazoles for the selective targeting of HDAC6.^94^ While this group has been known for several years, it was only recently that Christianson and Hansen showed DFMOs act as mechanism-based slow-binding inhibitors.^94,95^ Preliminary in vitro studies have shown successful brain penetrance, oral bioavailability, low metabolic clearance and an acceptable safety profile with no genotoxicity, or cardiotoxicity. Despite these benefits, DFMOs also present their own set of challenges, primarily concerning the chemical stability of the oxadiazole ring in the blood and non-neutral pH environments.

A third approach was the use of deacetylase inhibitors as synergizing therapeutics combined with standard-of-care or as adjuvant drugs following a primary treatment course.^96^ While HDACi have long been studied as single agents, they rarely provided sustained clinical benefits, in part due to the dose-limited efficacies and recurring toxicities. Alternatively, it was postulated that HDACi could help sensitize the epigenetic framework of a diseased cell (e.g., in cancer), allowing the standard-of-care to achieve better clinical benefits. These combinations have been shown to improve efficacy and reduce tumor resistance mechanisms, in addition to allowing smaller and less frequent doses of either medicine resulting in an overall safer dosing regimen.^97,98^ At the same time, combination strategies enable researchers to “rescue” current inhibitors instead of initiating new drug discovery programs.

On this front, HDACi have been explored across various human cancers, in combination with topoisomerase inhibitors (e.g., doxorubicin), kinase inhibitors (e.g., pazopanib), glucocorticoid antagonists (e.g., dexamethasone), immunotherapies (e.g., anti-PD1 Ab), radiotherapies, as well as other epigenetic drugs (e.g., 5-azacytidine). These studies are summarized in an excellent review by Hontecillas-Prieto et al.^99^ Furthermore, as epigenetic drug hunters turn their attention beyond oncology, HDACi have also been combined with antiretroviral therapeutics for HIV-1 treatment.^100,101^ These molecules reactivate latent viral reservoirs, allowing antiretrovirals to eliminate traditionally inaccessible virion populations and improve patient prognoses.^57^ Through this dual shock-and-kill approach, HDACi provide a promising avenue toward a potential curative therapy against HIV1, with several clinical trials reported in recent years (e.g., NCT03198559, NCT05700630, NCT03525730).^57^

In the clinic, after a decade without any new drug applications, givinostat was recently approved for the treatment of DMD—an incurable neuromuscular disorder, which primarily affects pediatric males (<6 years old).^53,102^ Despite its poor selectivity profile as an unselective HDACi, this approval represents an important milestone outside of cancer. Another great example is the CKD series out of Chong Kun Dang Pharmaceutical Corp. (CKD-504, CKD-510, etc.), currently under investigation for the treatment chronic neuropathies (e.g., CMT1A).^103^ While its structure is not disclosed, clinical candidate CKD-510 is a nonhydroxamate HDACi, highlighting this more recent move away from this suboptimal ZBG. Overall, with the increasingly growing list of HDACi under clinical investigation, it appears that the clinical landscape of these molecules may be on the cusp of a new paradigm outside of oncology, away from traditional structural designs and open to new pharmacological approaches.

## 3 Non-canonical Inhibitors: A New Paradigm?

The current limitations of HDAC inhibitors in oncology, as well as the search for therapeutic avenues in other diseases, has motivated the investigation of alternative strategies in HDAC inhibition. On the one hand, compounds with long target engagement, i.e., slow kinetics, have arisen as more efficient tools than the first generation of approved drugs. As a result, research is focusing on the kinetic optimization of reversible inhibitors and the development of irreversible covalent strategies. On the other hand, degradation with proteolysis-targeting chimeras (PROTACs) is regarded as a complementary strategy that may expand the scope of HDAC-targeting therapeutics.^104−107^ Driven by preclinical successes and first-in-human validation against other targets (e.g., ARV-110, ARV-471),^108^ the HDAC field has rapidly adopted degradation as an alternative. This endeavor has provided reports of >100 HDAC-PROTACs in literature, as it has been reviewed elsewhere.^109−111^

Similar to the interaction of membrane receptors with their ligands,^112^ HDACi interact with their targets to form both short- and long-lived complexes.^113^

The combined rate of dissociation of each binding pose confers an HDAC inhibitor with a characteristic residence time (τ), which determines the overall duration of the inhibition event.^114,115^ This dynamic aspect of inhibition is often overlooked during compound development, even though high τ values are often indicative of success in the clinic.^114,116^

**Figure 4:**
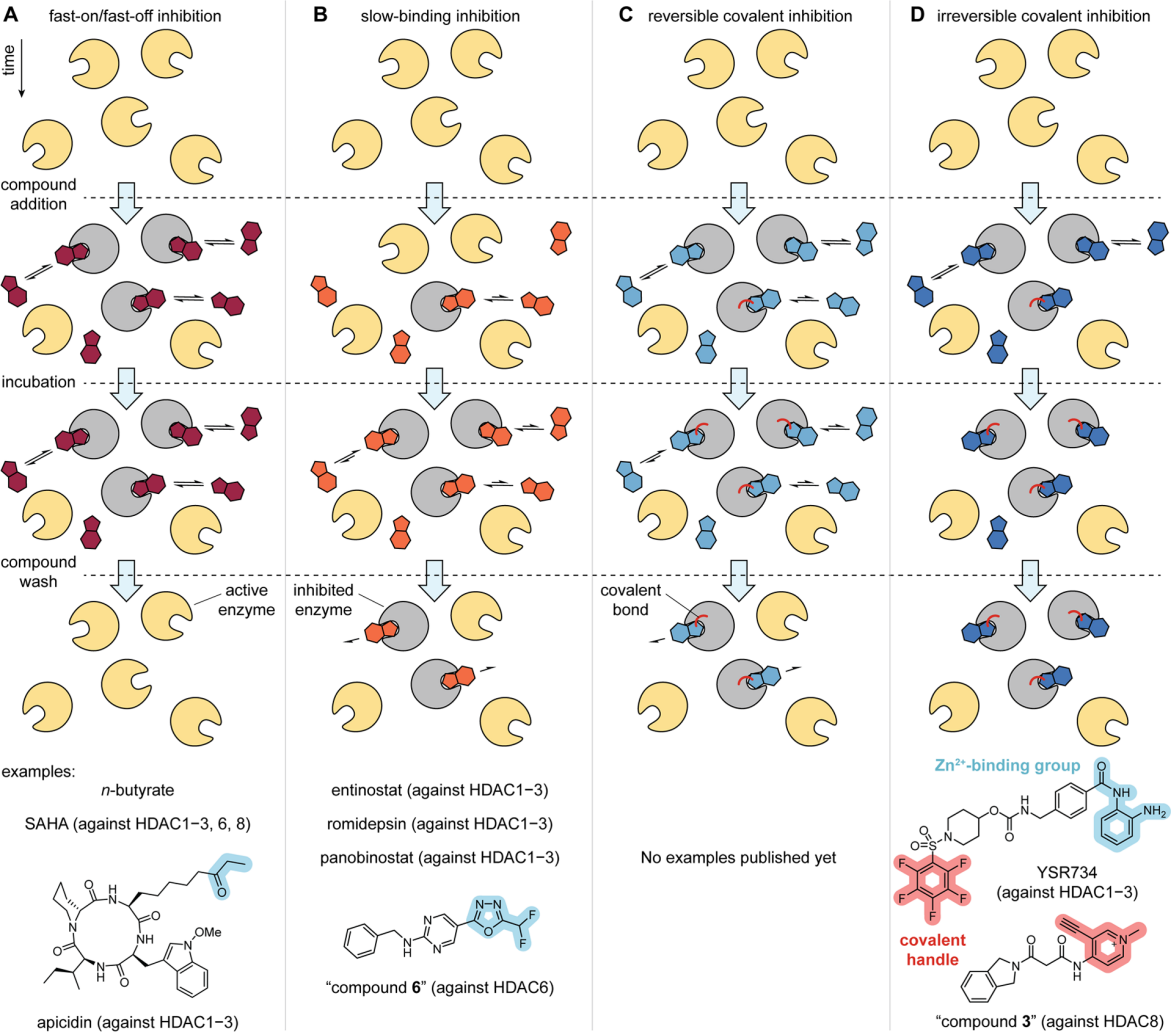
Schematic representation of target engagement after addition, incubation and wash of (a) fast-on/fast-off inhibitors, (b) slow-binding inhibitors, (c) reversible covalent inhibitors, and (d) irreversible covalent inhibitors, with examples of HDACi following each kinetic mechanism.

In standard end-point assays, inhibitors are assumed to display fast-on/fast-off kinetics (Figure 4A), where τ would typically be on the order of seconds or shorter. Under these circumstances, inhibitors instantly reach equilibrium with their targets, and any alteration of free-compound concentration is rapidly reflected on target occupancy (Figure 4A).^115^ This is the case for SAHA,^113^ and initially assumed for most small-molecule HDACi. On the contrary, early work from Gottesfeld and co-workers revealed that an *o*-aminoanilide SAHA derivative was a slow-binding inhibitor with τ of hours against HDACs 1–3, significantly extending the effect of this compound in cells.^113^ Subsequent kinetic studies revealed that most *o*-aminoanilides are slow-binding inhibitors (Figure 4B),^117^ including entinostat^3,4^ and compounds targeting the “foot pocket”.^118^ Since slow-binding inhibitors do not reach equilibrium instantly, their observed potency increases gradually during incubation. More importantly, concentration changes are not immediately reflected on target occupancy, due to their slow dissociation rates from the enzyme, which is highly attractive in an in vivo context (Figure 4B).^115^ Thus, continuous monitoring of activity and τ determination is essential to determine the potency and selectivity of slow-binding inhibitors, as reflected by the recent *o*-aminoanilide literature.^118−120^ Importantly, these compounds can display kinetic selectivity even between a free HDAC enzyme, the same HDAC in a multiprotein complex,^120^ and between different complexes.^121^

After the identification of *o*-aminoanilides as slow-binding inhibitors, many other chemotypes have been shown to display slow kinetics. A trifluoromethyl ketone version of SAHA,^122^ various alkyl hydrazides,^119,123^ and *o*-substituted benzamides,^124^ are all slow-binding inhibitors of HDACs 1–3. Slow inhibition of HDAC8 was achieved with trifluoromethyl ketone inhibitors,^125^ and of HDAC6 with DFMOs (compound **6**, Figure 4B).^95,126^

The natural products romidepsin and trapoxin A were found to be slow-binding inhibitors with τ of up to several hours, which serves to rationalize their excellent potency in vivo.^127−129^ Finally, the hydroxamic acid derivatives of apicidin and trapoxin, as well as the archetypal inhibitors panobinostat and TSA, are slow-binding inhibitors of subsets of class I, class IIb and class IV enzymes with different kinetic profiles of selectivity among them (Figure 4B).^128,129^

**Figure 5:**
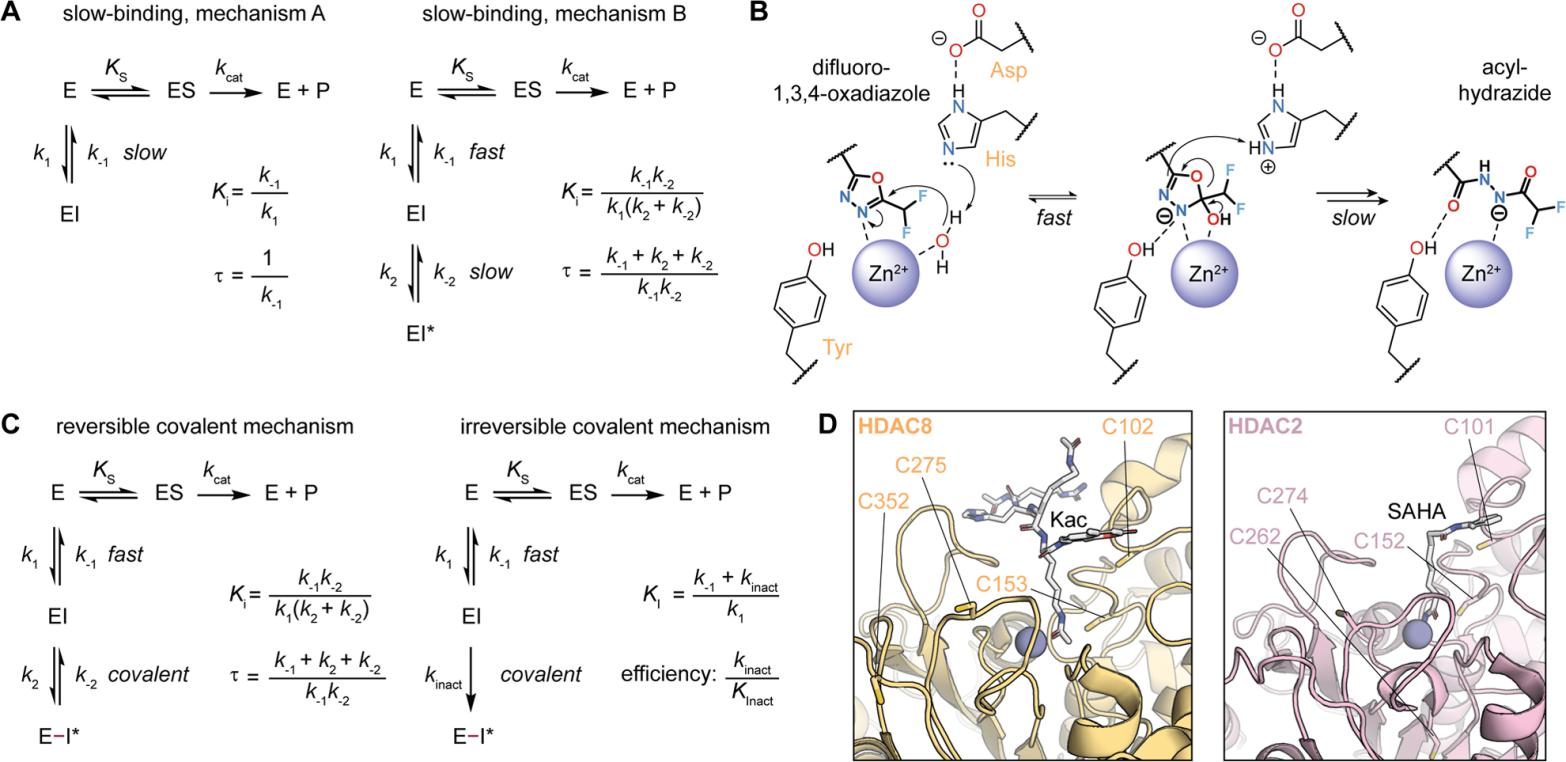
(a) Standard mechanisms of slow-binding inhibition, and calculation of inhibitor potency (*K*_i_) and residence time (τ).^1,2^ (b) Mechanism-based inhibition (as judged by the nucleophilic H_2_O attack) of HDAC6 by difluoro-1,3,4-oxadiazoles (DFMOs).^131−133^ (c) Standard mechanisms of covalent inhibition and calculation of inhibitor potency (*K*_i_) and residence time (τ) for reversible inhibitors or inactivation constant (*K*_I_, or *K*_Inact_, not to be confused with *K*_i_) and efficiency for irreversible inhibitors. (d) Cysteine residues nearby the HDAC8 (PDB 2V5W) and HDAC2 (PDB 4LXZ) active sites.

Even though most approved HDACi are now known to be slow-binding inhibitors, which highlights the potential of slow kinetics in the clinic, the lack of clear structure-kinetic relationships (SKR) hampers slow-binder design. Most slow-binding inhibitors follow the so-called “mechanism B” of target inhibition, where a first, fast-binding event is detected, followed by a transition to a more stable and long-lived enzyme–inhibitor complex (Figure 5A).^114,130^ The basis of this transition, often referred to as “induced-fit”, is unknown for most chemotypes, and thus difficult to exploit for SKR studies. Interestingly, recent studies on DFMOs have provided a first rational mechanism B transition, which does not entail an induced fit but rather a mechanism-based transformation. These compounds inhibit HDAC6 with high selectivity and τ of several hours, as the catalytic water molecule in the HDAC6 active site performs a nucleophilic attack on the outermost C=N bond of the oxadiazole and forms a tetrahedral intermediate coordinated to the Zn^2+^ ion (Figure 5B). The oxadiazole ring then opens to afford a hydrolyzed acyl-hydrazide, and this transition is rate-limiting and likely responsible for the slow-binding behavior.^131,132^

After that, the acyl-hydrazide dissociates or gets further deacylated to the free hydrazide, but none of these species show as high potency, selectivity, or τ as the original DFMO.^95,126,131−133^ This mechanism has thus served to rationalize the oxadiazole structural and electronic requirements to achieve slow kinetics.^131−133^ Unfortunately, such knowledge is still missing for other slow-binding HDAC inhibitor scaffolds.

A second strategy to extend the τ of a compound is to include a reactive handle for covalent bond formation. This approach is often more intuitive and easier to design, although it requires finding accessible reactive residues within the HDAC structure. Since the covalent interaction provides a large gain of affinity, this approach can also potentially remove the need for a strong Zn^2+^ chelator such as the hydroxamic acid. There are two possible mechanisms of covalent inhibition: (1) the formation of a reversible covalent interaction, where the inhibitor can be regenerated and dissociate from the enzyme (Figure 4C), and (2) an irreversible bond formation, where the fate of the complex would be the degradation of the HDAC and/or the inhibitor (Figure 4D).^134^ In both cases, the system can be modeled as a two-step process with a first fast binding step and a second slow covalent transition. In reversible covalent inhibition, the kinetic profile is similar to the mechanism B of slow kinetics, whereas irreversible covalent inhibition affords a system with theoretical infinite τ values (Figure 5C).

The first two reports of covalent HDAC inhibition were in 2015, on HDAC-targeted compounds that released Cys-reactive species. The laboratories of Yingjie Zhang and Wengfang Chu developed SAHA-based compounds with a phenylsulfonylfuroxan as a NO-donating functionality,^135^ which was also later incorporated into an HDAC6-targeting scaffold.^136^ The NO generated by these inhibitors is transferred to neighboring sulfides such as those of Cys residues on the surface and the catalytic pocket of HDACs.^135,136^ The laboratories of Seth Cohen and Carol Fierke found a similar compound class serendipitously, as their quinonyl-SAHA (SAHA-TAP) prodrug led to modification of multiple Cys residues of HDAC8 by the released quinone cap.^137^

Class I HDACs and, in particular, HDAC8 have reactive Cys residues on the surface surrounding the active site and within the substrate pocket. As the latter residues are relatively close to the Zn^2+^ ion (Figure 5D), they are thought to be involved in the sensing of electrophilic Lys modifications.^138^ Promiscuous reactive fragments can label both internal and surface residues,^125,139^ and they can be further developed into potent irreversible inhibitors. For instance, 3-ethynylmethylpyridiniums were developed as covalent HDAC8 inhibitors targeting Cys-containing allosteric sites (compound **3**, Figure 4D).^139^ This new approach permits targeting potentially unique pockets for isozyme- or complex-selective inhibition, and the development of potent HDAC inactivators without a Zn^2+^-binding group.^139^

HDAC1–3 display less surface Cys residues than HDAC8, but their distribution is similar around the active site (Figure 5D). These four enzymes share a reactive Cys residue 10 Å away from the entrance of the active site with a slight change in position in HDAC8 (HDAC8 C275 vs HDAC2 C274, Figure 5D).^140^ The addition of a Cys-reactive handle to the cap group of a class I inhibitor thus improves potency by further extending τ on these enzymes.^140^ This effect was recently shown by modifying the scaffold of the HDAC1–3 inhibitor entinostat with the irreversible pentafluorobenzenesulfonamide electrophyle (YSR734, Figure 4D), which adds covalent targeting to the three enzymes and provides longer lasting effects in cells.^140^ This proof-of-concept study opens up the possibility of maximizing τ with the addition of Cys-targeting moieties, and provides a framework for identifying enzyme-specific residues toward selective HDAC inactivation. Moreover, recent developments in protein covalent chemistry have expanded the possibilities beyond Cys targeting,^141−143^ which will facilitate the rational design of future covalent HDAC inhibitors.

## 4 THE BENEFITS OF IMPROVING INHIBITOR KINETICS

**Figure 6:**
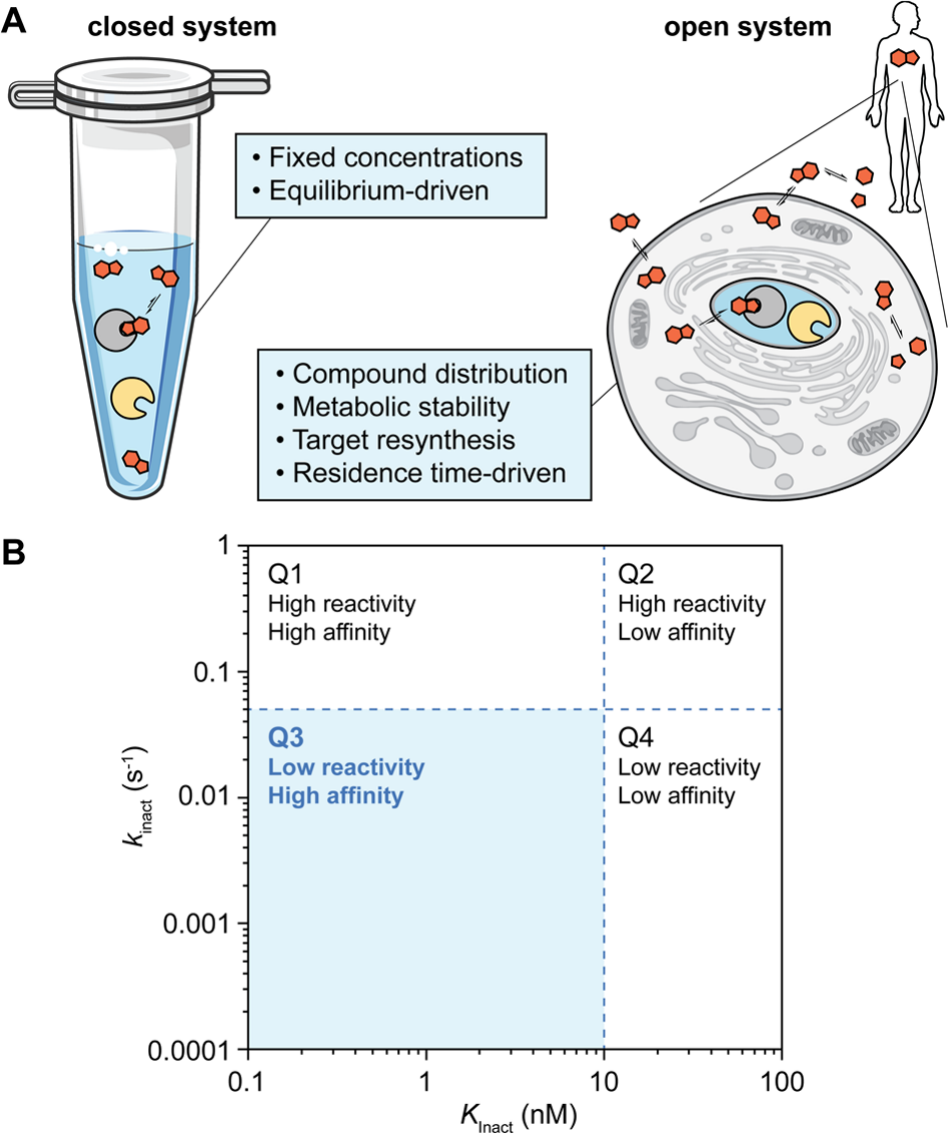
(a) Comparison of closed (laboratory experiment) and open (human body) systems, inspired by Copeland et al.^112^ Tube icon provided by bioicons.com. (b) Quadrant distribution of irreversible covalent inhibitor properties.

Traditionally, most (if not all) drug discovery programs quantify inhibition events by deriving dissociation constants (*K*_d_), inhibition constants (*K*_i_), or half-maximal inhibitory concentrations (IC_50_). Governed by thermodynamic equilibria, these macroscopic potency metrics operate within a simple closed system, which assumes invariable receptor–ligand concentrations. While useful for controlled in vitro assays, such a simplistic view of pharmacology breaks down in the complex milieu of a living cell (Figure 6A). In our pursuit of bioactive molecules with clinical benefits in human patients, medicinal chemists must try to adopt experimental models that most closely represent their target environment. In this regard, Copeland et al.^115^ promoted the use of the drug-target residence time as a central parameter in lead optimization programs, providing a measure of the duration of a drug’s action, and thus a more accurate insight into in vivo drug pharmacology.^115^

Optimizing residence time (τ), or in the case of irreversible inhibitors their *k*_inact_/*K*_I_, is an overall more promising strategy (Figure 5A, C), as [drug]_free_ in a cellular environment is in fact in constant flux due to competing mechanisms of absorption, permeation, metabolism, and excretion. Concurrently, [target]_free_ also varies due to competing substrates, protein resynthesis, and degradation pathways (Figure 6A).^114^ Achieving extended residence times ultimately results in the *decoupling* of pharmacodynamic (PD) profiles, as a slowly dissociating molecule can outlast its systemic stability (i.e., half-life) or the protein resynthesis rates.^130^ Thus, slow-binding and covalent inhibitors hold better promise toward achieving smaller and less frequent dosing regimens, as required for most HDACi. Taken to their clinical extreme, these benefits could offer better safety profiles, reduced costs, and improved compliance.

For slow-binding and reversible covalent inhibitors, optimizing τ entails primarily the optimization of their off-rate (Figure 5A, C). Interestingly, Copeland also highlights the importance of on-rates, which can drive target selectivity as well as the subtle phenomenon of drug rebinding. As ligands dissociate from their target, a high local concentration is generated at the binding site interface, which without any rapid clearing mechanism enables rebinding and the extended pharmacological effect. For irreversible covalent inhibitors, there is no off-rate and thus τ cannot be calculated. Instead, the ratio of the covalent bond formation kinetics (*k*_inact_) and the inactivation constant (*K*_I_, or *K*_Inact_, not to be confused with *K*_i_) serves as a dynamic measure of the compound’s efficiency (Figure 5C). Here, typical thresholds of *k*_inact_ = 0.05 s^–1^ and *K*_Inact_ = 10 nM would define desirable kinetics (Figure 6B), with lead compounds laying under the *k*_inact_ threshold (Q3). These insights are lost when measuring broad thermodynamic metrics. While previously challenging to ascertain, dynamic parameters can now be regularly measured via kinetic binding experiments through, for example, NMR or surface plasmon resonance, or with continuous enzyme activity assays using fluorogenic substrates.^144^ In addition, studies are proposing new expedited approaches to estimate these important metrics.^145,146^ When supported with pharmacokinetic AUC_free_ values, these data would allow the estimation of in vivo target occupancy, which can help guide covalent dosing regimens.

The true extent of sustained residence times and decoupled PK–PD has been regularly exemplified in the rich field of covalent drugs.^147−150^ With >50 covalent drugs currently on the market, >15 FDA-approvals in the past 10 years alone and an annual market cap estimated at >US$50 billion, covalent molecules have firmly positioned themselves as arguably one of the most successful clinical strategies to date.^151^ Covalent inhibitors are known for their powerful potencies (pM–nM range), sustained target engagement (even in the presence of endogenous substrates), and a unique ability to engage intractable drug targets (e.g., flat, solvated, allosteric pockets).^152^ Previous concerns of nonspecific hyperreactivity have been largely resolved with recent advances in chemoproteomic protein profiling, soft electrophiles with tunable reactivities, and an overall better understanding of the required benchmarks for a successful preclinical/clinical covalent drug candidate.^134,149,150,153,154^

Regarding targeted HDAC binders, researchers have started to explore the potential benefits of slow-binding or covalent kinetic mechanisms. Shifting to a long-lived (or permanent) binding strategy would disengage inhibitor PD from any inherent PK liabilities and provide more sustained pharmacology.^155^ While several slow-binding HDACi have been reported in recent literature, we still lack an explicit and predictable framework of the structure-kinetic forces underlying these mechanisms. Thus, it is clear that these are complex and multifactorial processes with important contributions from both the ligand and target protein. For instance, simple hydroxamic acids were long presumed to represent a classic case of potent ligands with fast-on/fast-off kinetics, while bulkier ZBGs such as *o*-aminoanilides were assumed to be needed for slow-on/slow-off kinetics, due to structural rearrangements in the protein active site (i.e., break an internal H-bond).^117^ Instead, recent reports have demonstrated that small hydroxamic acids and alkyl-hydrazides can also be slow-binding inhibitors.^129,156^ On the other hand, covalent inhibitors represent a more mature field of study, with defined structure–activity trends, established design principles and relatively predictable molecular recognition. Several HDAC isozymes have also been shown to have nucleophilic residues within targetable distances of their catalytic domains, as seen in studies on HDAC2 (YSR734), HDAC6 (SAHA-TAP), and HDAC8 (Compound **3**).^137,140,157^ At this early stage, these molecules remain as useful chemical starting points and further optimization efforts are needed to effectively probe the potential benefits of irreversible HDACi binders. As the field compares the preclinical tractability of reversible, slow-binding, or covalent molecules, detailed research into current structure-kinetic trends would be a great step toward improving HDAC targeting.

## 5 Conclusion

Dysregulated acetylation mechanisms are an established hallmark of human disease. Intensive research into these signatures ultimately led to the discovery and classification of a family of deacetylases with Zn^2+^-dependent activity (HDACs 1–11). With cell-wide influence on post-translational acylation of proteins and acetylation of polyamines, these hydrolase enzymes have established themselves as master regulators of cell homeostasis.^50^ At the same time, uncontrolled HDAC activity has broad implications in cellular dysfunction and disease progression, including cancer, neurodegeneration, inflammation and infection. Thanks to early phenotypic results and extensive crystallographic work, HDACs are now useful drug targets with a wealth of inhibitors reported, generally acting as competitive acetyl-lysine mimetics.^4^ However, besides a handful regulatory approvals, the clinical adoption of first gen. HDACi (e.g., SAHA, belinostat, panobinostat) is far from widespread, and it is largely confined to small patient populations with specific disease states. Arguably, the causes of these limitations are the nonspecific inhibitory profiles and the use of warheads with harmful metabolites, which afford dose-limited efficacies and recurring clinical toxicities.

To address limitations in HDAC targeting, several paradigms shifts have occurred toward the next generation of therapeutics, namely isozyme-specific inhibition, non-hydroxamate ZBGs, combination therapies and targeted protein degradation.^10^ Along with these strategies, recent literature has seen a notable rise of the novel kinetic mechanisms of inhibition of slow-binding kinetics and covalent targeting. These mechanisms hold promise in achieving more sustained efficacies in vivo and better pharmacokinetic profiles, through extended drug-target residence times. To accomplish this, studies have explored substituted *o*-aminoanilides,^8^ alkyl hydrazides,^158^ mechanism-based difluoromethyl-1,3,4-oxadiazoles,^95^ quinonyl pro-drugs,^137^ ethynylmethylpyridiniums,^139^ and irreversible perfluorinated arylsulfonamides.^140^ Interestingly, recent reevaluations revealed the slow-binding properties of successful first gen. HDACi, further supporting this approach.

While great strides have been made and the field is in the midst of a transformation, these studies remain in their early stages.^159−161^ Future efforts on this front will likely focus on establishing reproducible structure-kinetic relationships, delineating isozyme-specific slow-binding and covalent strategies, and establishing tractable preclinical and clinical guidelines for better slow-binding and covalent inhibitor design. All in all, we envision that HDACi with improved kinetic profiles hold promise toward extending the clinical benefits of targeting HDACs in human disease.

## ABBREVIATIONS

AD: Alzheimer’s disease
CMT: Charcot-Marie-Tooth
CTCL: cutaneous T-cell lymphoma
DFMO: 2-(difluoromethyl)-1,3,4-oxadiazoles
DMD: Duchenne muscular dystrophy
HDAC: histone deacetylase
HDACi: HDAC inhibitor
HIV: human immunodeficiency virus
MM: multiple myeloma
PD: pharmacodynamics
PK: pharmacokinetics
PROTAC: protein-targeting chimera
PTCL: peripheral T-cell lymphoma
PTM: post-translational modification
ZBG: Zn^2+^-binding group
